# PATTERNS OF PERFORMANCE ON QUALITY OF CARE AMONG U.S. HOME HEALTH AGENCIES, 2015-2017

**DOI:** 10.1093/geroni/igz038.1831

**Published:** 2019-11-08

**Authors:** Chenjuan Ma

**Affiliations:** New York University, New York, New York, United States

## Abstract

Home healthcare is a critical care source for community-dwelling older adults. As the fastest growing healthcare sector in the US, quality of home healthcare is under increasing scrutiny. The purpose of this study is to examine patterns of performance on quality of care among US home health agencies. This is a 3-year cohort study using 2015-2017 Home Health Compare data and Provider of Services (POS) Files. In the dataset, each HHA was assigned a star rating (1-5) to reflect the overall quality of care. This indicator was calculated based on two process measures (timely initiation of care and drug education) and six outcome measures (e.g., hospitalization). We examined 8,020 HHAs in the US. Over the 3-year period, the number of HHAs receiving a star rating of 4 or 5 increased from 27% in 2015, 31% in 2016, to 32% in 2017. Roughly, 32% of the HHAs received a lower star rating and another 32% received a higher star rating from 2015 to 2016. Similarly, 30% of the HHAs received a lower star rating and 29% of the HHAs received a higher star rating from 2016 to 2017. Hospital-based HHAs were less likely to receive a star rating of 4 or 5. Larger HHAs (OR 1.34; 95% CI, 1.13-1.59) and HHAs with ownership changes (OR, 1.38; 95% CI 1.20-1.59) were more likely to improve their star ratings overtime. Our finding indicates dynamic changes in the quality of care within the US home healthcare sector.

